# Modulation of Nrf2 by Olive Oil and Wine Polyphenols and Neuroprotection

**DOI:** 10.3390/antiox6040073

**Published:** 2017-09-26

**Authors:** Miriam Martínez-Huélamo, Jose Rodríguez-Morató, Anna Boronat, Rafael de la Torre

**Affiliations:** 1 Integrated Pharmacology and Systems Neuroscience Research Group, Neurosciences Research Program, IMIM-Institut Hospital del Mar d’Investigacions Mèdiques, Dr. Aiguader 88, 08003 Barcelona, Spain; mmartinez4@imim.es (M.M.-H.); jrodriguez1@imim.es (J.R.-M.); aboronat@imim.es (A.B.); 2 Department of Experimental and Health Sciences, Universitat Pompeu Fabra (CEXS-UPF), Dr. Aiguader 80, 08003 Barcelona, Spain; 3 CIBER de Fisiopatología de la Obesidad y Nutrición (CIBEROBN, CB06/03/028), 15706 Santiago de Compostela, Spain

**Keywords:** polyphenols, Nrf2, wine, olive oil, cognitive function, resveratrol, hydroxytyrosol

## Abstract

Strong adherence to a Mediterranean diet is associated with improved cognitive function and a lower prevalence of mild cognitive impairment. Olive oil and red wine are rich sources of polyphenols which are responsible in part for the beneficial effects on cognitive functioning. Polyphenols induce endogenous antioxidant defense mechanisms by modulating transcription factors such as the nuclear factor (erythroid-derived 2)-like 2 (Nrf2). This review discusses the scientific data supporting the modulating effect of olive oil and red wine polyphenols on Nrf2 expression, and the potential health benefits associated with cognitive functioning.

## 1. Health Benefits of the Mediterranean Diet: Potential Contribution of Dietary Phenolic Compounds

The traditional Mediterranean diet (MD) is characterized by a high intake of plant-based food: fruit, vegetables, legumes, nuts, and non-refined cereals. Moderate consumption of dairy products, poultry, and fish, and low intake of red meat is recommended. Olive oil, as a main source of fat, and moderate alcohol drinking (mainly in the form of red wine) are specific foods of this dietary pattern [1].

MD in the context of the PREDIMED (Prevención con dieta Mediterránea) nutritional intervention clinical trial has been shown to be cardioprotective [2] and to have an overall ameliorating effect on older adults’ global cognition, with a lower incidence of mild cognitive impairment (MCI) [3,4]. Meta-analyses of clinical studies have only partially confirmed these results, mainly because the heterogeneity in design and methodologies.

An interesting observation made in the PREDIMED study concerning cognition was that the increased consumption of antioxidant-rich foods in general and of polyphenols in particular was associated with better cognitive performance in elderly subjects at high cardiovascular risk [5]. This is of relevance because MD is not only constituted by an adequate balance of nutrients, but also by a number of biologically active non-nutrient substances. Among them, phenolic compounds are major non-nutrient substances in our diet, mainly in plant-based foods (fruit, vegetables, cereals, legumes, and chocolate) and beverages (red wine, cocoa, and coffee). It is estimated that the total dietary intake of phenolic compounds is 1 g/day [6].

### 1.1. Olive Oil Polyphenols 

Virgin olive oil (VOO) is the main source of fat in the MD and contributes considerably to the health properties attributed to this diet [7]. Traditionally, the benefits of VOO were attributed to its high monounsaturated fatty acid content, principally oleic acid. However, through randomized, crossover, and controlled trials such as the EUROLIVE study, it is now acknowledged that these effects should also be credited to the phenolic fraction of VOO, rich in minor bioactive compounds, that can provide benefits for plasma lipid levels and oxidative damage [8,9]. According to the European Food and Safety Authority (EFSA), there is a cause-effect relationship between the intake of olive oil phenolic compounds (standardized for the content of tyrosol, hydroxytyrosol (HT), and derivatives) and the protection of blood lipids from oxidative stress. In order to obtain these beneficial effects, EFSA established a minimum daily intake requirement of 5 mg of HT and its derivatives [10].

The major components of olive oil represent 98–99% of the total weight of the oil and are formed mainly of triacylglycerides. Amongst the fatty acids that constitute the triacylglycerols, monounsaturated oleic acid is the main representative (55–83%), followed by polyunsaturated (4–20%), and saturated fatty acids (8–14%).

On the other hand, the minor components of olive oil make up only around 1–2% of the total weight of the oil, but include more than 230 chemical compounds [11]. Phenolic compounds can be found in this group, and, due to their potential beneficial health effects, are the most studied and best-known components [12,13]. Triterpenes constitute another group belonging to the minor components of olive oil and, as a result of their bioactive properties, are also the focus of research. They include the pentacyclic compounds oleanolic acid, maslinic acid, ursolic acid, betulinic acid, uvaol, and erythrodiol [14,15].

**Figure 1:**
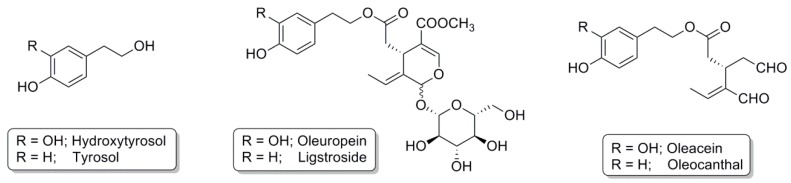
Major biologically active phenolic compounds present in olive oil.

The phenolic composition of VOO varies considerably (50–940 mg/kg) as it is strongly affected by multiple factors including the cultivar, area of origin, age of the olive tree, degree of ripeness, soil composition, climate, processing technique, and storage [8,16]. Amongst the VOO phenolic compounds that have attracted scientific interest due to their beneficial effects are the following: HT, tyrosol, oleuropein, ligstroside, oleacein, and oleocanthal (Figure 1) [17,18,19].

### 1.2. Wine Polyphenols

**Figure 2:**
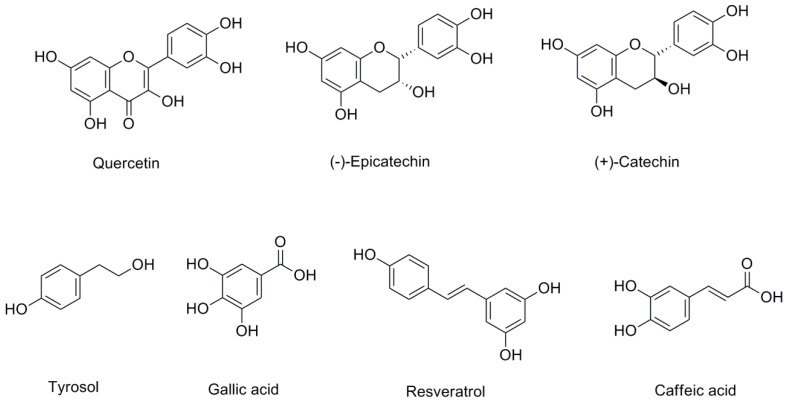
Representative examples of wine phenolic compounds with biological activities.

Wine is an alcoholic beverage made of the fermented must, or juice, pressed from grapes. To date, more than 500 compounds have been identified in grapes and wine. The major constituents of wine are water (80–90%) and ethanol (8–15%), followed by carbon dioxide, glycerol, sugars, polysaccharides, higher alcohols, acids, and phenolic compounds. Whilst wine phenolic compounds originate primarily from the grapes, they are also produced during winemaking and maturation [20]. During fermentation, a process mediated by yeasts, sugars are transformed into ethanol and carbon dioxide. At the same time, the chemical composition of phytochemicals present in grapes is modified, giving rise to more than 200 phenolic compounds, including both flavonoids (the main group) and non-flavonoids [21]. Quercetin, (−)-epicatechin, (+)-catechin, tyrosol, gallic acid, resveratrol, and caffeic acid are representative examples of wine phenolic compounds that, as explained below, have attracted interest due to their biological activities (Figure 2).

The amount of phenolic compounds present in wine is highly variable and depends on the grape source, variety, and processing. Red wines are richer in phenolic compounds than white ones due to the fact that they are obtained by the alcoholic fermentation of the musts in the presence of seeds and skins. In contrast, white wines are exclusively produced by the fermentation of the grape juice (the seeds and skin are quickly separated after the pressing) [22,23]. In general terms, red wine contains around 10-fold more phenolic compounds than white: the total phenolic content in white wine ranges from 190 to 290 mg/L, whereas in red wine it varies from 900 to 2500 mg/L [21].

### 1.3. Bioavailability of Phenolic Compounds Including Their Ability to Cross the Blood Brain Barrier

The bioavailability of wine phenolic compounds has been reviewed recently [24]. Major phenolic compounds from wine (i.e., gallic acid, catechin, epicatechin, and metabolites) have been identified in rats plasma following acute gavage; however, only repeated dosing resulted in brain deposition of metabolites [25]. In humans, there are indirect evidences of the brain bioavailability of catechins. In a clinical trial in multiple sclerosis, measuring changes in brain *N*-acetyl-aspartate (NAA) after the administration of green tea extracts using a point resolved magnetic resonance spectroscopic imaging sequence, it was demonstrated that they correlated with plasma concentrations of epigallocatechin gallate (EGCG) [26]. We stated earlier that the bioavailability of dietary phenolic compounds was poor. This is due to the metabolism of these compounds in the gut by microbiota, poor absorption in some cases, and more importantly because of an extensive first pass metabolism in the intestinal epithelium and in the liver. The resulting metabolites, mainly sulfate, methylated, and glucuronide conjugates, were supposed in the past to be biologically inactive. There are several examples of conjugates that still retain some biological activity, while others are in some tissues bio-transformed in a way that the free form is released from its conjugate moiety [27,28]. In vitro studies show that metabolites may cross the blood brain barrier (BBB), and thus become bioavailable in the brain [29]. Available data is still scarce, but future studies will provide evidence that phenolic compounds’ metabolites contribute to the overall health benefits elicited after their intake.

Previous discussions have mainly focused on flavonoids present in wine; in the case of olive oil, simple phenolic compounds such as HT are more relevant. The metabolic clearance of HT and other simple phenols of dietary origin have been reviewed recently [19]. In rats, HT is distributed through the blood stream to the best-irrigated tissues, including the brain; therefore, it is able to cross the BBB even if brain concentrations are low [30].

It is relevant to mention that the maintenance of a high plasma concentration of polyphenols and their metabolites as well as their bioavailability in the brain requires repeated dosing. This requirement is of relevance in the context of dietary patterns.

### 1.4. Mechanisms of Action of Phenolic Compounds

From a chemical point of view, phenolic compounds are molecules with a phenyl ring bearing one or more hydroxyl groups. The presence of a phenolic group capable of reducing reactive oxygen species (ROS) confers redox properties, and in vitro, most of these compounds display strong antioxidant activities [23]. For this reason, phenolic compounds have traditionally been considered useful in the prevention of chronic diseases associated with oxidative stress (e.g., cardiovascular and neurodegenerative diseases, diabetes, and cancer). In vitro antioxidant effects of phenolic compounds are probably of relevance in plants, for fighting against stressful environmental conditions, as well as in the gut, where relatively high concentrations can be achieved after the ingestion of food. In vivo, in humans, the poor bioavailability of these compounds and the low concentration achieved in tissues and biological fluids discounts the idea of a direct antioxidant mechanism to explain the health benefit effects derived from their consumption. Nevertheless, clinical studies have reported that the beneficial effects of MD foods like olive oil are dependent on the amount of phenolic compounds ingested [9].

The ability of phenolic compounds to interact with biological systems modulating, for example, gene expression has been one of the mechanisms postulated explaining part of health benefits [31,32]. New mechanisms have mainly been studied in animal models and in vitro, and include interaction with intracellular signaling pathways (Nrf2, nuclear factor-κB, MAP kinase, and PI3 kinase/Akt) [33,34], the activation of endothelial nitric oxide production and release, interference in anti-apoptotic processes, regulation of amyloid β peptide synthesis and degradation [35,36], reduction of tau aggregation [37], and reduction of neuroinflammation [24], as well as the modulation of gene expression [34,38], microRNAs [39], and proteins [40], amongst others.

In the present review, we focus on phenolic compounds from olive oil and wine, key components of the MD, and examine the extent to which beneficial effects seen in cognitive function associated with their consumption could be explained in part by their ability to modulate the nuclear transcription factor (erythroid-derived 2)-like 2, also known as Nrf2 (or NFE2L2), that plays a pivotal role in regulating the expression of antioxidant proteins that protect against oxidative damage. Preclinical studies suggest that this could be the case [41].

## 2. Nuclear Factor (Erythroid-Derived 2)-Like 2 (Nrf2) and Neurodegenerative Diseases

### 2.1. Oxidative Stress as a Trigger for Neurodegenerative Diseases

Free radicals are atoms and molecules that have a single unpaired electron in an atomic or molecular orbital. These unpaired electrons cause high reactivity to free radicals, triggering oxidative stress when there is an over- or unbalanced production of oxidized/damaged macromolecules that are not efficiently removed and renewed [42,43]. The principal class of generated radical species is ROS. They can be defined as oxygen-containing molecules or fragments with one or more unpaired electrons in atomic or molecular orbitals [44,45,46]. ROS generation can originate through external sources such as ultraviolet rays, high-energy irradiation, xenobiotic metabolism, and the redox-cycling of quinones, ions, metals, and nitroaromatics [42,47,48]. Nevertheless, approximately 90% of ROS are produced in the mitochondria respiratory chain complex [49,50]. ROS damage cell membranes and increase their rigidity, leading to modifications in proteins, lipids, and cell membrane receptors. With respect to the latter, this alters their function, intracellular signaling, and clustering with other membrane receptors [51]. Oxidative stress and ROS production can result in cellular damage when constructing the molecular basis, and thus can also result in the progression of a number of neurodegenerative diseases [52,53,54,55,56].

### 2.2. Nuclear Factor (Erythroid-Derived 2)-Like 2 (Nrf2) as a Protector of Neurodegenerative Processes

Human antioxidant defense is made up of enzymatic and non-enzymatic components capable of reducing the damage caused by free radicals by quenching them before they react with biologic targets, chelating redox metals, interacting with other antioxidants, having a positive effect on gene expression, and preventing chain reactions and the activation of oxygen to highly reactive products [45,57]. One of the most important cellular defense mechanisms against oxidative stress is the Nrf2-antioxidant response element (ARE) pathway. Nrf2 is a member of the family of transcription factors that activates a battery of cytoprotective genes through the ARE, participating in biotransformation, antioxidant reactions, and inflammation [58,59,60,61,62,63]. These genes encode a large variety of enzymatic components such as NAD(P)H quinone oxidoreductase-1 (NQO1), UDP-glucuronosyltransferases, glutamate cysteine ligase, glutathione peroxidase, thioredoxin, thioredoxin reductase, peroxiredoxin, heme oxygenase-1 (HO-1), glucose-6-phosphate dehydrogenase, glutathione S-transferase (GST), sulfiredoxin 1 (SRXN1), malic enzyme 1, members of the aldo-keto reductase (AKR), ferritin, heat shock proteins, metallothionein, and multidrug resistance-associated proteins [64]. Several studies have also indicated a relationship between Nrf2 and NFĸB, a transcription factor responsible for inflammatory response, suggesting that the regulation of redox homeostasis by Nrf2 could lead to the modulation of NFĸB activity. Under normal conditions, Nrf2 is maintained inactive in the cytoplasm bound to the BTB-Kelch-like ECH-associated protein 1 (Keap1), a receptor of electrophilic compounds and stimulator of Nrf2 ubiquitination and proteosomal degradation. The Nrf2/Keap1 complex dissociates when Keap1 is modified, inducing the access of Nrf2 to the nucleus where it forms a heterodimer with one of the small Maf proteins which binds the ARE and activates transcription to initiate antioxidant response [60,61,65,66]. Several Nrf2 regulation forms have been described in the literature. The best known is the Keap1 dissociation by oxidative stress or by the covalent modification of the thiol groups of Keap1. Post-translational modifications, for example, serine and threonine phosphorylation by various kinases such as phosphatidylinositol 3-kinase (PI3K), protein kinase C (PKC), c-Jun N-terminal kinase (JNK), and protein kinase regulated by extracellular signals (ERK) have also been proposed as Nrf2 activators [67,68,69].

Nowadays, Nrf2 has generated increasing interest since it has been demonstrated to provide protection against some neurodegenerative diseases, although there is a controversy concerning which cell type activates Nrf2. Depending on the neurodegenerative disease studied, it has been seen that different cells can be activated. In Parkinson’s disease, Nrf2 is activated in astrocytes; in Alzheimer’s disease in astrocytes, neurons, and microglia; and in multiple sclerosis in all glial cells but not in neurons. Further evidence is required to determine in which cells Nrf2 activation occurs in response to treatments targeting Nrf2. This will provide insight into the molecular mechanisms driving disease and opportunities for the treatment of neurodegeneration [70].

Here, we will briefly summarize the main studies performed evaluating the potential role of Nrf2 on various neurodegenerative diseases such as Alzheimer’s, Parkinson’s, Huntington’s, multiple sclerosis, and cerebral ischemia. Although Nrf2 is activated by several types of compounds, either of natural (dietary) origin or synthetic compounds, the following examples will only focus on bioactive compounds derived from some specific foods.

#### 2.2.1. Alzheimer’s Disease (AD)

Worldwide, Alzheimer’s disease is the most common neurodegenerative disorder in the elderly, with nearly 44 million affected in 2016 [54,55]. AD symptoms cause problems with memory, thinking, vision/spatial issues, and impaired reasoning and judgment [54]. Several research lines have demonstrated that oxidative stress plays a detrimental role in AD, leading to nucleic acid, lipid, and protein damage, in addition to a reduction in the antioxidant enzymatic barrier [42,43,53,55,71,72]. The causes that may increase ROS levels and their accumulation in AD include: mitochondrial dysfunction, high levels of β-amyloid peptide, transition metal accumulation, and genetic factors [43,56,71]. Nrf2 was shown to take part in the reduction of oxidative stress in AD in several in vitro and in vivo studies, helping in this way to reduce the disease progression. The transcription factor increased HO-1 expression in Aβ1-42-treated mice. This enzyme exerts anti-neuroinflammatory effects and protects neurons against neurotoxin-induced cell death, ameliorating cognitive deficits in mice [73]. Zhou et al. [74] demonstrated that Nrf2 regulates the expression of HO-1, GST, and aldo-keto reductases 1C1 and 1C2, thus inhibiting ROS accumulation and reducing oxidative stress damage in HT-22 hippocampal cells. The neuroprotective effect of Nrf2 was also evaluated by Wang et al. [75] in SH-SY5Y cells treated with Aβ25–35, which produces neurotoxic activity. Nrf2 was able to induce HO-1 expression, reducing the viability loss and apoptotic rate, and attenuating Aβ-mediated ROS production.

#### 2.2.2. Parkinson’s Disease (PD)

PD is the second most common neurodegenerative disorder after AD, and affects 10 million people worldwide. PD is characterized by the development of tremors, slower and more rigid muscle movements, and the reduction of reflexes contributing to a loss of balance. As the disease gradually worsens, many secondary symptoms develop, such as depression, anxiety, emotional changes, cognitive impairment, difficulty swallowing, chewing, and speaking, masked facial expressions, urinary problems, constipation, fatigue, and sleep problems [54,66,71,76]. Although the exact mechanism remains unclear, several aspects could explain this disease: genomic factors, epigenetic changes, toxicity, oxidative stress, neuroimmune/neuroinflammatory reactions, hypoxic-ischemic conditions, metabolic deficiencies, and ubiquitin–proteasome system dysfunction. Oxidative stress has been particularly considered as one of the essential mechanisms in PD. Several studies have shown that it oxidizes lipids, proteins, and DNA, reduces antioxidant enzymes and non-enzymatic antioxidants levels, increases mitochondrial DNA mutation, lowers reduced glutathione (GSH) and increases oxidized GSH levels, alters protein homeostasis, and modulates dopamine release in PD patients. It has also been reported that the reduced activity in Complex I of the respiratory chain of these patients may contribute to ROS generation and induce apoptosis [54,55,56,66,77,78].

A number of studies have been published asserting the protective effect of the Nrf2 pathway in PD. Jing et al. [59] demonstrated the neuroprotective effect of Nrf2 against 6-hydroxydopamine (6-OHDA)-induced neurotoxicity in human neuroblastoma SH-SY5Y cells. Nrf2 upregulated HO-1, GSH cysteine ligase regulatory subunit, and GSH cysteine ligase modulatory subunit in the cells.

In a similar manner, a study on 6-OHDA-induced dopaminergic neuronal death in vivo and in vitro was performed. Nrf2 may be involved in the upregulation of HO-1 protein in glial cells and provide a beneficial effect against the dopaminergic neuronal loss caused by 6-OHDA [79]. Cui et al. [80] investigated the protective effect of Nrf2 against rotenone-induced dopaminergic neuronal oxidative damage in a rat model of PD. Nrf2 activated HO-1 and NQO1 levels, increased GSH levels, and reduced endogenous oxidative markers (ROS and malondialdehyde).

#### 2.2.3. Huntington’s Disease (HD)

HD is a hereditary condition resulting from mutation of the Huntington gene located on chromosome 4 (4p63). HD causes irritability, depression, small involuntary movements, poor coordination, and trouble learning new information or making decisions [53,54,81,82,83]. Significant scientific evidence suggests oxidative stress to be a progressor of HD. The principal mechanisms involved in the ROS-mediated etiopathogenesis of the disease are impairment in the electron transport chain and mitochondrial dysfunction [54]. Several studies have reported increased levels of lipid peroxidation by-products, high values of compounds resulting from protein carbonylation and nitration, oxidation of both mitochondrial and nuclear DNA, and modified activity/levels of some enzymatic antioxidants in HD patients [54,82,84]. As for the two previously mentioned neurodegenerative diseases, there are also several research lines that have studied the role of Nrf2 in the progression and development of HD. The Nrf2/ARE pathway could enhance the transcription of target genes coding for antioxidant enzymes, and an elevation of the levels of antioxidant compounds. Sandhir et al. [85] induced HD with 3-NP, decreasing Complex II activity in rats. Nrf2 enhances the GSH levels and superoxide dismutase (SOD) activity, together with the reduction in mitochondrial swelling, lipid peroxidation, protein carbonyls, and ROS showing a significant improvement in neuromotor coordination. The beneficial effect of Nrf2 was analyzed in striatal cells derived from HD knock-in mice expressing mutant Htt versus wild-type striatal cells. Nrf2 enhanced SOD1 mRNA and SOD activity, as well as glutamate-cysteine ligase catalytic subunit mRNA and protein levels [86]. Other evidence was given by Gao et al. [87]; they reported that Nrf2 acted against 3-NP-induced oxidative stress in a rat model of HD, activating the expression of HO-1 and NQO1 in striatum [87]. Stack et al. [88] employed N171-82Q mice, a transgenic mouse model of HD, to study Nrf2-related genes. The transcription factor could reduce ROS, induce NQO1, HO1, and glutathione S-transferase alpha 3 (GST-α3) genes in the brain and peripheral tissues, reduce oxidative stress, increase longevity, improve the motor impairment and striatal atrophy in the brain, and vacuolate in brown adipose tissue.

#### 2.2.4. Multiple Sclerosis

Multiple sclerosis (MS) is a chronic inflammatory disease of the central nervous system affecting approximately 2.5 million people worldwide [89,90]. Genetic, immunological, and environmental factors are involved in the development of this condition. It is associated with inflammation and oxidative stress, inducing lesions of white and gray matter with cortical demyelination. Higher levels of oxidative stress markers and lower antioxidant capacity have been found in MS patients. Some mechanisms have been proposed to explain the role of ROS in MS neurological damage including: (i) low levels of antioxidants that stimulate transendothelial leukocyte migration through the BBB breakdown, thus contributing to immunoinflammatory processes in the cerebral cortex; and (ii) excess ROS increasing T cell concentration and producing myelin damage [56,90,91,92]. Nrf2 is also involved in the progression of MS, although studies with dietary compounds are scarce. One example was reported by Zhang et al. [93], who evaluated the protective effect of Nrf2 on myelin sheath destruction in a cuprizone-induced demyelination model in mice. Nrf2 enhanced HO-1 and NQO1 expression levels, ameliorating hyperlocomotion and impairment. On the other hand, there have been several studies performed with synthetic compounds, such as dimethyl fumarate, capable of activating Nrf2 and consequently the expression of Nrf2 target genes. NQO1 and HO-1 gene expression were also demonstrated to be activated by Nrf2 following ex vivo stimulation of human peripheral blood mononuclear cells (PBMCs) [94]. Wang et al. [95] utilized mouse and rat neuronal stem/progenitor cells (NPCs) and neurons to detect an Nrf2 neuroprotective effect. Nrf2 reduced ROS production and, consequently, decreased oxidative stress-induced apoptosis and promoted the survival of motor neurons under oxidative stress.

#### 2.2.5. Cerebral Ischemia

Ischemic cerebrovascular disease is one of the leading causes of death worldwide. About 75% of those who survive a cerebral ischemia have their quality of life affected due to several degrees of disability or the complete loss of self-care abilities. Brain ischemia emerges when a reduction of cerebral flow appears due to a transient or permanent thrombotic occlusion of a major cerebral artery. Whilst the mechanism by which the disease develops is still unclear, it has been observed that various mechanisms are interrelated and affect one another [96]. The accumulation of excitatory neurotransmitters (mainly glutamate), an increase in intracellular Ca^2+^, the activation of several enzymes including phospholipases, cyclooxygenases, nitric oxide synthases, and proteolytic enzymes, the release of ROS which triggers lipid peroxidation, DNA injury, and mitochondrial dysfunction are frequent in cerebral ischemia patients. All these situations promote the activation of several transcription factors such as NFκB, hypoxia-inducible factor 1 (HIF-1), and STAT3, and consequently the production of inflammatory cytokines, adhesion molecules, and the recruitment of activated leukocytes culminating in a local tissue inflammation [53]. Nrf2 has been described as one of the possible mechanisms involved in the improvement of cerebral ischemia consequences. Several authors have reported its beneficial effect in in vivo and in vitro models. Nrf2 increased the content of antioxidant enzymes and reduced the generation of oxidative species after experimental cerebral ischemic/reperfusion injury in mice. The activation of the enzymes improved neurological scores, reduced infarct volume, and attenuated cellular apoptosis [97]. Kuo et al. [98] investigated Nrf2 in ischemic stroke in a mouse model. There was a reduction of infarct size, brain edema, lessened BBB disruption, and ameliorated neurological deficits attributed to the Nrf2 pathway. Yang et al. [99] demonstrated the improvement of neurological deficits and the reduction of the infarct size through the Nrf2 pathway in a transient rat middle cerebral artery occlusion model.

## 3. Modulating Effect of Olive Oil and Red Wine Polyphenols on Nrf2 and Neurodegenerative Diseases

Higher adherence to the MD is associated with improved cognition and a reduced risk of neurodegenerative diseases [100,101,102,103]. Dietary phenolic compounds have been related with these beneficial health effects and are considered to be, in part, responsible for its neuroprotective activities [5,6]. A number of these compounds are able to modulate the Nrf2/ARE pathway and thus represent a potential tool for the prevention of cognitive decline and neurodegenerative diseases [41]. As has been previously described, the Nrf2/ARE pathway is intimately involved in the pathogenesis of neurodegenerative diseases, being a potential neuroprotective factor [61] and a target to modulate neuroinflammation [104]. Some examples of dietary phenolic compounds that are known to exert neuroprotection via Nrf2 include the yellow pigment in curcumin [41] and the green tea flavanol EGCG [105].

### 3.1. Olive Oil

A large body of research has accumulated with respect to olive oil, the primary source of dietary fat in the MD. Olive oil has been reported to have health benefits for cardiovascular and neurodegenerative diseases due to the presence of phenolic compounds in its matrix [14,19]. The principal phenolic compounds are HT, tyrosol, and oleuropein, which play a key role in the deceleration of neurodegenerative disease development and progression (Table 1). Some of these studies assign these beneficial effects to the activation of the Nrf2 pathway.

#### 3.1.1. Hydroxytyrosol

HT, which in olive oil is found at a concentration of approximately 1.9 mg/kg (we refer here to its free from; it is mainly present in the oleuropein secoiridoid chemical structure), is one of the phenolic compounds most researched [14]. Several studies have demonstrated its beneficial effects, using both in vitro and in vivo models, and its multiple biological characteristics such as antioxidant activity, cancer cell growth inhibition, apoptosis induction, and cardiovascular and neuronal protection [106]. Although, to date, the mechanisms of action remain unclear, some authors have demonstrated that HT is able to activate the Nrf2 pathway. As has been seen in previous sections, most neurodegenerative diseases have oxidative stress in common, which provokes inflammation and the deterioration of neuronal cells. The activation of Nrf2 by HT could be an effective strategy to protect cells against possible damage caused by oxidative stress. In this regard, several in vitro studies have been developed. Bigagli et al. [107] investigated the anti-inflammatory effect of HT and oleuropein at physiological concentrations (5–10 μM) in macrophages RAW264.7 stimulated with lipopolysaccharide (LPS), which induced inflammation. The two compounds inhibited intra- and extracellular oxidative stress in a dose-dependent manner, with HT as the most active [107]. The effect was corroborated by Visioli et al. [108], who reported a stronger radical scavenging activity for HT than oleuropein. Nrf2 activation was also evaluated, and HT was able to activate Nrf2 translocation to the nucleus, thus contributing to the activation of the Nrf2/ARE pathway. Inhibition of indicators of oxidative stress with the activation of Nrf2 was therefore correlated [107]. Likewise, HT (10–50 µM) protects neuron-like rat pheochromocytoma cells (PC12) from hydrogen peroxide-induced death and 6-OHDA-induced damage scavenging free radicals, and shields cells from free radical-induced oxidative damage. Moreover, HT activates the Nrf2/ARE pathway, leading to the upregulation of protection enzymes from these toxic molecules, such as glutamate-cysteine ligase, HO-1, NQO1, and thioredoxin reductase [106]. PC12 cells were also studied in the alteration of dopamine after HT treatment. The intra-neuronal enzymatic metabolism of dopamine passes through the intermediate metabolite, 3,4-dihydroxyphenylacetaldehyde (DOPAL), which contributes to the loss of dopaminergic neurons in PD. Monoamine oxidase inhibitors (MAO-I)—used in the treatment of Parkinson’s—decrease DOPAL production, but at the same time build up cytoplasmic dopamine, resulting in increased spontaneous oxidation to dopamine-quinone and the formation of potentially toxic compounds, including 5-S-cysteinyl-dopamine (Cys-DA). An overproduction of Cys-DA might offset the beneficial effects of decreasing DOPAL production. For this reason, PC12 cells were incubated with HT alone or with MAO inhibitors. Results showed that HT inhibits both enzymatic and spontaneous oxidation of endogenous dopamine and mitigates the increase in spontaneous oxidation during MAO inhibition [109]. Another study demonstrated the protective effect of HT on the IMR-32 human neuroblastoma cell line after methyl mercury (MeHg)-induced neurotoxicity. HT reduced MeHg-induced cellular oxidative stress along with the maintenance of GSH, SOD, glutathione-S-transferase, and catalase. HT (5 µM) downregulated p53, bax, cytochrome C, and caspase 3, and upregulated Nrf2 and metallothionein. HT promotes the expression of Nrf2, which, in turn, elevates the expression of various cellular GSH/GST levels, thereby ameliorating the adverse effects of MeHg [110].

The protective effect of HT has also been demonstrated in animal models, corroborating the results obtained in vitro. Tasset et al. [111] demonstrated the brain antioxidant effect of HT against the oxidative stress caused by 3-nitropropionic acid (3NP) in Wistar rats after the administration of 2.5 mg/kg body weight HT for 14 days. 3NP caused a rise in lipid peroxides and a reduction in GSH content. After HT treatment, lipid peroxidation was reduced and GSH depletion was blocked, attributed to the activation of the Nrf2 pathway [111]. A noteworthy study was performed by Zheng et al. [112] investigating the HT preventive effect on prenatal stress-induced behavioral and molecular alterations in offspring. It is well known that prenatal stress induces emotional and cognitive dysfunction in the offspring of both humans and experimental animals. For this reason, rats were exposed to restraint stress during days 14–20 of pregnancy. HT was given at doses of 10 and 50 mg/kg/day. After exposing rats to stress, both male and female offspring presented impaired learning capacity and induced memory performance. The effects were reestablished in the HT supplement groups with the prevention of the stress-induced downregulation of neural proteins. Oxidative stress and mitochondrial dysfunction were confirmed in stressed offspring with changes in protein oxidation such as SOD, the expression of mitochondrial complexes, and mitochondrial DNA copy number. Meanwhile, HT significantly increased transcription factors, as well as phase II enzyme-related proteins, including Nrf2 and HO-1, decreased oxidative stress, and augmented mitochondrial function. Taken together, these findings suggest that HT could be used not only to correct the deterioration of neurodegenerative diseases, but also to prevent neurogenesis and cognitive function in prenatally stressed offspring by activating the Nrf2 pathway [112].

The beneficial effect of HT in animal and in vitro models has been demonstrated in the studies previously described, but unfortunately, there is a lack of clinical investigations. Only one study with HT has been performed in humans, and the results were not satisfactory. Crespo et al. [113] tested the effect of HT on phase II enzymes’ expression activated through the Nrf2 pathway in a double-blind, randomized, placebo-controlled study. Two doses were tested, 5 and 25 mg/day orally administration (Hytolive^®^, an olive mill waste water extract enriched in HT, (Genosa, Madrid, Spain)) for one week. HT did not significantly modify phase II enzyme expression in peripheral blood mononuclear cells, although the bioavailability of the HT preparation is unknown. It is essential to perform human studies to corroborate whether the results obtained in cells and animals with HT have the same beneficial effects derived from Nrf2 activation.

#### 3.1.2. Tyrosol

Tyrosol is another phenolic compound found in olive oil as well as in some alcoholic beverages such as beer, wine, and champagne. It has been reported that the antioxidant potential of tyrosol is weaker compared to its hydroxyl form, HT, probably due to the absence of the ortho-diphenolic group in its chemical structure. In spite of its diminished antioxidant strength, several experiments have focused on its biological activities and reported protection against many pathological conditions including neurodegenerative diseases. Tyrosol was evaluated in a transient, middle cerebral artery occlusion rat model regarding infarct volume and sensory motor function deficit after ischemia. It demonstrated a dose-dependent neuroprotective effect in treated rats (10–30 mg/kg of tyrosol) and protective effects against sensory motor dysfunction. Tyrosol reduced the infarct volume and improved the neurological outcome. Although the precise neuroprotective mechanism is still unclear, these findings imply that tyrosol passed the BBB and showed antioxidant and anti-inflammatory effects in the brain, leading to this neuroprotection [114]. As previously reported for HT, tyrosol showed a protective effect for PD. Vauzour et al. [115] investigated in mouse cortical neurons the potential neuroprotective effects of tyrosol, *p*-coumaric acid, and caffeic acid (0.1–50 µM) on CysDA-induced neuronal injury. They compared their results with flavonoid components such as epicatechin, catechin, and quercetin. Significant protection was reported for *p*-coumaric and caffeic acids, and tyrosol was equal to or greater than those observed for the flavonoids. Specifically, tyrosol displayed higher protection than catechin. The neuroprotective effect of tyrosol (50–200 µM) was also evaluated in catecholaminergic neuron cells after the induction of neuronal death with the parkinsonian toxin 1-methyl-4-phenylpyridinium (MPP+) by Dewapriya et al. [116]. MPP+ enters the dopaminergic neurons through dopamine transporters and blocks the mitochondrial electron transporter chain, thus producing oxidative radicals in neurons, which ultimately cause neuronal death. Tyrosol exerted neuroprotection through the attenuation of the mitochondrial dysfunction and intracellular ATP depletion in a dose- and time-dependent manner. The upregulation of antioxidant proteins, such as SOD-1, SOD-2, and DJ-1, was also revealed.

Although the effect of tyrosol has been demonstrated, its mechanism regarding neurodegenerative diseases remains to be solved. Other research areas have, however, described that the Nrf2 pathway is activated by tyrosol in in vivo and in vitro models. One example of this was the work performed by Wang et al. [117], who studied the effect of tyrosol on the expression of HO-1 and Nrf2 activation in C57BL/6 male mice treated with LPS, the predominant pathogen which induces acute lung injury (ALI) and acute respiratory distress syndrome (ARDS). The results showed that, in the LPS group and after treatment with tyrosol, HO-1 expression was improved and Nrf2 was activated. This suggests that tyrosol can upregulate HO-1 through the Nrf2 pathway in LPS-induced ALI.

In summary, the mechanisms of action of tyrosol in neurodegenerative diseases warrant further research. According to the previous articles reviewed, tyrosol has been found to display a protective role at a neuronal level by preventing neuronal death in PD cells and reducing infarct volume. Moreover, it has been reported to improve neurological outcome following transient focal cerebral ischemia in rats; its mechanism of action is, nevertheless, still unknown. A number of studies have reported that tyrosol is a regulator of the antioxidant activity of Nrf2. It is possible that this mechanism could also be involved in neurodegenerative diseases in a similar manner to HT and other phenolic compounds such as resveratrol in wine, curcumin in curcuma, and epigallocatechin-3-gallate in green tea [107,112,118].

#### 3.1.3. Oleuropein

The bitter taste of immature and unprocessed olives is due to oleuropein, the principal glycoside. Oleuropein has been reported to display protective effects against acute cardiotoxicity, and anti-ischemic and hypolipidemic activities. It has also recently been shown to provide neuroprotection for several degenerative diseases including AD and PD. Such neuroprotection has been attributed to the formation of a non-covalent complex with the Aβ peptide, which is a key trigger of AD [119]. In double transgenic TgCRND8 mice, as a model of amyloid-β deposition, Grossi et al. [120] evaluated the effects of an eight-week dietary supplementation of oleuropein aglycone (50 mg/kg of diet). Oleuropein improved the cognitive performance of mice by reducing β-amyloid levels and plaque deposits. Further research performed by the same group extended the study to aged TgCRND8 mice that typically present higher pE3-Aβ deposits in the brain. Oleuropein treatment (50 mg/kg of diet) counteracted glutaminylcyclase-catalyzed pE3-Aβ generation, reducing enzyme expression and interfering both with Aβ42 and pE3-Aβ aggregation. Such effects, even at a late stage, suggest that the polyphenol could be beneficial at both therapeutic and preventive levels for AD [121]. Another study evaluated the dose-response effect of diet supplemented with oleuropein (12.5/0.5 mg/kg) and a mix of polyphenols found in olive mill waste water (50 mg/kg); both improved the cognitive functions of TgCRND8 mice. Aβ42 and pE-3Aβ plaque number and area were reduced in the cortex by oleuropein, and in the cortex and hippocampus by the dietary polyphenol mix [122]. The ability of oleuropein to react against Aβ proteotoxicity was evaluated in *C. elegans*, as a simplified invertebrate model of AD. *C. elegans* treated with oleuropein presented reduced Aβ plaque deposition, less abundant toxic Aβ oligomers, decreased paralysis, and increased lifespan [123].

The PC12 cell model has been used to investigate oleuropein effects in the PD 6-OHDA-induced neurotoxicity model in several studies. Oleuropein incubation (20 and 25 µg/mL) decreased cell damage and reduced biochemical markers of cell death, suggesting an antioxidant protective effect against 6-OHDA-induced PC12 cell damage [124]. Oleuropein is also capable of preventing neuronal degeneration in a PC12 cell model by reducing mitochondrial ROS production and blocking SOD activity [125].

Additionally, it is able to offset glutamate-induced neurotoxicity in HT-22 hippocampal neuronal cells, which has been reported to lead to PD and AD. Glutamate treatment has been observed to cause neuronal cell death and the translocation of mitochondrial apoptosis-inducing factor to the cytoplasm of HT-22 cells. Moreover, it increased mitochondrial fragmentation and dysfunction. After oleuropein treatment, translocation of mitochondrial apoptosis-inducing factor to the cytoplasm was inhibited. There was also a modification in the glutamate-induced mitochondrial dynamic imbalance and a reduction of the number of cells with fragmented mitochondria [126].

The mechanisms involved in neurodegenerative diseases for the beneficial effects of oleuropein are still unclear, as in the case of tyrosol. Nevertheless, there is scientific evidence that Nrf2 could be activated by oleuropein in cardiovascular disease models, and that these observations may be translated to neurodegenerative diseases. Parzonko et al. [127] examined whether oleuropein (1–10 µM) could protect endothelial progenitor cells (CD31^+^/VEGFR-2^+^), responsible for the neovascularization of ischemic tissue, against impairment of their functions due to angiotensin-induced cell senescence. Oleuropein-treated cells showed an increase in proliferation and telomerase activity, and a decrease in senescent cells and ROS levels. Oleuropein could restore migration, adhesion, and tube formation of endothelial progenitor cells in a concentration-dependent manner. Nrf2 and HO-1 expression were also evaluated, obtaining an increase in HO-1 expression and an activation of Nrf2 after oleuropein treatment. Sun et al. [128] assessed oleuropein antihypertensive and antioxidant functions in the paraventricular nucleus (PVN) for hypertension. In order to carry out the study, the authors used spontaneously hypertensive rats treated with 60 mg/kg/day of oleuropein. After eight weeks of oleuropein administration, the compound reduced blood pressure, pro-inflammatory cytokines, and the expression of components of the renin-angiotensin system. In addition, there was an increase in the antioxidant defense system compared to the control group. They also found a higher mitochondrial biogenesis and more regulated mitochondrial dynamics. Furthermore, Nrf2, NQO-1, and HO-1 were all increased in the PVN of the oleuropein-treated group compared with the control group. On the basis of their results, the authors demonstrated that oleuropein can protect the PVN from oxidative stress by improving mitochondrial function through the activation of the Nrf2 pathway.

### 3.2. Wine

#### 3.2.1. Resveratrol

Resveratrol has attracted considerable interest due to its potential neuroprotective characteristics. It can trigger beneficial effects in neurological and neurodegenerative diseases by attenuating oxidative stress. Resveratrol is able to directly scavenge free radicals and activate the endogenous antioxidant system. One of its potential targets is Nrf2 activation.

The effect of resveratrol has been studied in vitro with different neurological cell models. Resveratrol pretreatment (1–20 µM) on neuronal stem cells activated Nrf2 and protected the cells from oxygen-glucose deprivation/reoxygenation, which is used to mimic cerebral artery occlusion and reperfusion injury. Nrf2 protein levels were increased, thus upregulating the expression of Nrf2 target genes such as NQO-1 and HO-1. Moreover, the activity of SOD and GSH in the pretreated cells increased in a dose-dependent manner and malondiadehyde (MDA) was reduced [129]. Piceatannol (10 µM), a resveratrol metabolite, was used in HT22 neuronal cell cultures in order to study its effect on the modulation of oxidative stress. The expression and activity of HO-1 via Nrf2 activation were increased, suggesting that the metabolite is able to protect HT22 neuronal cells against glutamate-induced cell death, at least in part, by inducing Nrf2-dependent HO-1 expression [130]. Astrocytes are particularly rich in Nrf2, and their main function is the antioxidant modulation of neurons. Preconditioning of astrocytes with resveratrol activated Nrf2 and significantly increased protein levels of NQO-1. Resveratrol contributes to neuroprotection through maintaining mitochondrial coupling and antioxidant protein expression by the Nrf2 pathway [131].

A number of the studies were performed in vitro; moreover, there is also evidence from several studies focused on resveratrol neuroprotection in animal models in which Nrf2 plays a key role. In a pre-clinical study, rats were supplemented with resveratrol (15 and 30 mg/kg) for a week and afterwards a cerebral ischemic injury was induced. Resveratrol supplementation activated Nrf2, upregulating HO-1 expression. Moreover, resveratrol treatment decreased oxidation biomarkers and reestablished SOD activity. Overall, these changes were translated into better neurological scores and a decrease in the severity of the injuries [132]. Gaballah et al. [133] elucidated the mechanisms preventing neuronal death in a rat model of rotenone-induced PD after treatment with resveratrol. The stilbene (20 mg/kg) ameliorated rotenone-induced endoplasmic reticulum stress, restored redox balance suppressing xanthine oxidase activity and protein carbonyls formation, and activated Nrf2, which in turn activated glutathione peroxidase, preserving the intracellular antioxidants status. Resveratrol neuroprotective effect was also evaluated at postnatal day 7 in rat pups with fetal alcohol spectrum disorders after the administration of 100 mg/kg. Resveratrol prevented ethanol-induced apoptosis by scavenging ROS in the external granule layer of the cerebellum and increased the survival of cerebellar granule cells. It restored ethanol-induced changes in Nrf2 in the nucleus and retained the expression and activity of NADPH quinine oxidoreductase 1 and superoxide dismutase in the cerebellum of ethanol-exposed pups [134].

Endothelial function plays a major role in ensuring correct cerebral blood flow; its dysfunction is an early event in the pathogenesis of neurodegenerative diseases [135,136]. Resveratrol is able to activate Nrf2 and upregulate its driven antioxidant enzyme expression in coronary arterial endothelial cells, thus attenuating oxidative stress. In mice presenting endothelial dysfunction, resveratrol treatment could restore normal endothelial function and decrease ROS production. This effect was linked to the activation of Nrf2 due to the fact that mice, with an overproduction of Keap1 or Nrf2 knock-out, did not benefit from resveratrol treatment [137]. Resveratrol improvement of endothelial function via Nrf2 activation could be an indirect mechanism to induce neuroprotection.

The neuroprotective effect of resveratrol through Nrf2 activation has been demonstrated in vitro and in animal models, but studies describing this effect in humans are not available. Only one study was found where the protective effect of resveratrol is demonstrated in AD patients, but the research was not focused on the activation of Nrf2. One gram of resveratrol twice daily during 52 weeks decreased cerebrospinal fluid MMP9, modulated neuro-inflammation, and induced adaptive immunity in 119 subjects with mild-moderate AD [138]. Although the beneficial effect has not been linked to the Nrf2 pathway, it is a good starting point to continue in the investigation of this compound in humans.

On the other hand, many studies on inflammation diseases have been published in recent years. The postprandial effects of a supplement containing mainly resveratrol (100 mg) and grape polyphenols were studied in healthy volunteers. The supplement was administered within the context of a high-fat diet as an inductor of oxidation and inflammation. The intake of the supplement counteracted the postprandial oxidative and inflammatory stress. After its intake, Keap-1 expression was downregulated, promoting the translocation of Nrf2 to the nucleus and the expression of its driven target genes, NQO-1 and glutathione S-transferase P1 (GST-P1). Moreover, the supplement reduced pro-inflammatory biomarkers [139]. The effect of resveratrol (500 mg/day) on Nrf2 in non-dialyzed chronic kidney disease (CKD) patients was also evaluated. The results in this study were not satisfactory since the concentration of resveratrol administrated to the volunteers had no antioxidant or anti-inflammatory effect in this kind of patient [140].

#### 3.2.2. Epicatechin and Catechin

Whilst catechin, epicatechin (EC), as well as their oligomeric compounds procyanidines, have been reported to protect from oxidative stress, the molecular mechanisms involved have yet to be described. Nrf2 has emerged as a potential target.

In vitro studies have shown that the treatment of cortical astrocytes with EC (100 nM) increased Nrf2 levels, upregulating GSH [141]. EC (100 µM) triggered neuroprotection to embryonic cortical neuronal cells by decreasing oxidative stress and promoting cell viability. This effect was not observable in cells from Nrf2 and HO-1 knock-out mice [142]. The role of EC in neuroprotection has also been studied in mouse models of brain ischemic injury. EC supplementation (30 mg/kg) administered before the ischemic injury ameliorated its physiopathology, reducing the size of the damage, and the associated neurological deficits. Supplementation after the injury also improved the consequences of the brain infarct. As previously stated, these benefits were not noticeable in knock-out mice for Nrf2 and HO-1. This finding suggests that EC may exert its neuroprotection via the activation of Nrf2 and its stimulation of HO-1 activity [142]. In a similar manner, the administration of procyanidine (10–40 mg/kg), the oligomeric form of catechin and EC, to transient middle cerebral artery occlusion rats provided protection from induced brain ischemia, decreasing the damage triggered by the injury. Moreover, in the affected area of the brain, there was a significant reduction of ROS and MDA. Procyanidine administration restored Nrf2 levels and increased the protein expression of HO-1, GSTα, and NQO1 in the ischemic brain area [143]. A zebrafish model prone to developing tau aggregation, a hallmark of the pathogenesis of many neurodegenerative diseases, was supplemented with an herbal extract, the major compound of which was EC. There was a significant reduction in the formation of tau aggregates, and EC (60 mg/mL) was able to suppress their neurotoxicity through the activation of Nrf2 [144].

#### 3.2.3. Quercetin

Quercetin promotes the DNA binding of Nrf2 through two different mechanisms. On one hand, it enhances Nrf2 transcription and stabilizes Nrf2 at a posttranscriptional level, thus inhibiting its ubiquitination. On the other hand, quercetin promotes the degradation of Keap1. Overall, the Nrf2/Keap1 ratio increases, raising the nucleus Nrf2 concentrations, and effectively activating its interaction with the ARE [145].

Primary cerebellar granule neurons were pretreated with quercetin before an oxidative insult. Quercetin (25 µM) activated Nrf2 and may prevent neuronal death. Moreover, it raised GSH levels by means of an increase in the expression of glutamate-cysteine ligase catalytic subunit (GCLC), the rate-limiting enzyme in GSH synthesis [146]. In a study using a mouse model for traumatic brain injury, quercetin (50 mg/kg) exerted neuroprotection, attenuating the associated oxidative injury. Cytoplasmic Nrf2 decreased with quercetin supplementation, whereas nuclear Nrf2 augmented. The authors hypothesized that the protective effect of quercetin could come from the promotion of the translocation of Nrf2 to the nucleus more than an increase in its expression, although further research needs to be performed to elucidate the exact mechanism by which quercetin exerts its neuroprotection [147]. Recently, Dong et al. [148] also demonstrated quercetin neuroprotection in D-galactose-induced neurotoxicity in mice. Quercetin administration, 20-50 mg/kg, improved learning and memory, prevented changes in the neuronal cell morphology and apoptosis in the hippocampus, as well as increased the expression of Nrf2, HO-1, and SOD. On the other hand, when Nrf2 was inhibited, the effects of quercetin on HO-1 and SOD were reversed, as was the neuronal cell protection. Table 2 shows the main studies described above.

#### 3.2.4. Human Studies with Wine

Wine is a complex matrix containing a wide range of potentially active phenolic compounds at low concentrations, all of which may act synergistically to exert a beneficial effect. The effect of wine intake on Nrf2 activation in humans has not been described. However, several randomized, controlled, clinical trials administering wine measured the levels and activity of Nrf2-driven enzymes. After red wine consumption, there was an increase in the activity of glutathione peroxidase [149] and glutathione reductase [150,151]. These two enzymes are essential to maintain the GSH cycle and its correct functionality. GSH is the main antioxidant in brain cells. The dysregulation of its cycle has been related to the onset and progression of neurodegenerative diseases [152].

## 4. Conclusions

The MD, a dietary pattern characterized by a high intake of plant-based food, has been associated with a reduced prevalence of MCI and enhanced cognitive function [5,102,153,154,155]. Such beneficial effects have been attributed to a large variety of bioactive constituents, including phenolic compounds. Recently, olive oil, the main source of dietary fat in the MD, and red wine polyphenols have attracted researchers’ attention due to their potential role in the prevention of neurodegenerative diseases [14]. Although the review is focused on the study of the dietary polyphenols from olive oil and wine, we also want to highlight other by-products from these two foods since it has recently been demonstrated that they also contain high concentrations of bioactive compounds, particularly phenolic compounds that would be useful in the preparation of nutraceutical products [156,157].

Several neuroprotective mechanisms of action of phenolic compounds have been proposed, including antioxidant activity, regulation of amyloidosis, reduction of tau aggregation and neuroinflammation, and, more recently, interaction with intracellular signaling pathways, particularly Nrf2 [24,33,34,35,36,37]. Nrf2 is one of the most important cellular defense mechanisms against oxidative stress and is involved in the protection against some neurodegenerative diseases (AD, PD, HD, MS, and cerebral ischemia) by activating a battery of cytoprotective genes through interaction with the ARE [71,72,73,74,75,85,86,87,97,98,99]. It has been demonstrated that, in some cases, oxidative stress is not enough to activate Nrf2—external stimulation of the transcription factor is, therefore, necessary to carry out its protective function [158]. In this regard, several studies have been developed in in vitro and in animal models to assess different phenolic compounds as stimulators of the Nrf2 pathway. In this review, we have critically analyzed the current evidence of the effect of phenolic compounds found in wine and olive oil on cognitive function, with a special focus on the modulation of Nrf2. Some limitations to the studies reviewed are related to a lack of evidence of causality between phenolic compound exposure and neuroprotective effect modulated by Nrf2. The determination of secondary biomarkers of its activation is not enough. The use in experimental protocols of knock-out Nrf2^−/−^ animal models or siRNA techniques (i.e., [74,75,106]) would definitively provide the degree of scientific evidence missing in some reports.

Hydroxytyrosol, tyrosol, and oleuropein are the principal olive oil phenolic compounds which can exert beneficial effects in in vitro and animal models against the etiology and pathogenesis of many neurodegenerative diseases. In particular, hydroxytyrosol stimulates the Nrf2 pathway, allowing the reduction of oxidative stress indicators, dopamine, lipid peroxidation, and GSH depletion. These effects lead to an increment in mitochondrial function and phase II enzyme-related proteins, such as HO-1, which are factors that are related to neurodegenerative diseases [106,107,109,112]. In a similar manner, tyrosol is able to prevent neuronal death in PD cells, reduce infarct volume, and improve the neurological outcome following transient focal cerebral ischemia in rats [114,116]. In addition, oleuropein improved the cognitive performance of mice by reducing β-amyloid levels and plaque deposits. It also presented less abundant toxic Aβ oligomers, decreased paralysis, and increased lifespan in *C. elegans* as an AD model [120,123]. Although the effects of tyrosol and oleuropein have been demonstrated, the mechanism by which they act in neurodegenerative diseases is still unclear. However, their capacity as Nrf2 activators has been shown in other conditions, such as the effect of tyrosol on C57BL/6 male mice with acute lung injury and acute respiratory distress syndrome [117], and the influence of oleuropein in cardiovascular disease models [127].

Red wine presents more than 200 phenolic compounds, the most representative being resveratrol, catechin, epicatechin, and quercetin. Resveratrol is able to scavenge free radicals and can also activate the endogenous antioxidant system by the Nrf2 pathway, demonstrating a beneficial effect in neuronal cells and animal models. Resveratrol through Nrf2 activation upregulates the expression of NQO-1 and HO-1, increases SOD and GSH, and suppresses cerebral ischemic injury [129,130,131,132]. The activation of Nrf2 by resveratrol has also been reported in endothelial cells and mouse models, attenuating oxidative stress, decreasing ROS production, and restoring normal endothelial function, which is also linked to the pathogenesis of neurodegenerative diseases [137]. In the case of epicatechin and catechin, Nrf2 has emerged as a possible mechanism of protection, there is, however, less evidence than in the case of resveratrol. Epicatechin and catechin have demonstrated neuroprotection in in vitro studies upregulating GSH levels, decreasing oxidative stress, and promoting cell viability. Their beneficial effect was also observed in mouse models, reducing damage size and associated neurological deficits after ischemic injury [142]. Finally, quercetin promotes the degradation of Keap1 and induces the nuclear translocation of Nrf2. Additionally, it enhances Nrf2 transcription and stabilizes Nrf2 at a posttranscriptional level, inhibiting its ubiquitination [145].

On the basis of the current evidence, further studies are needed to clarify the mechanisms involved in neurodegenerative diseases, since the benefits attributed to olive oil and wine polyphenols are similar to those compounds that have shown these properties through the Nrf2 pathway. Additionally, despite the large body of evidence describing the activation of Nrf2 in vitro and in animal models, the majority of the previously mentioned studies employed concentrations and doses far greater than those commonly found in the diet. Whilst such studies are useful to understand mechanisms of action, their in vivo extrapolation should be performed with care, and studies in humans are necessary to corroborate the results.

## Figures and Tables

**Table 1:** Olive oil polyphenols and their potential involvement in neurodegenerative diseases.

Compound	Dose	Model/Treatment	Results	Reference
Hydroxytyrosol	5–10 µM	Macrophages RAW264.7 treated with LPS	Activation Nrf2 at 10 µM; Correlation Nrf2 with indicators of oxidative stress	[107]
	2.5 mg/kg	Wistar rats treated with 3NP	Reduction lipid peroxidation; Blocking GSH depletion	[111]
	10–50 µM	PC12 cells treated with H_2_O_2_ and 6-OHDA	Activation Nrf2; Upregulation of glutamate–cysteine ligase, HO-1, NQO1 and thioredoxin reductase	[106]
	10 µM	PC12 cells treated with MAO inhibitors	Decreased levels of DOPAL and Cys-DA—Prevention of an increment in Cys-DA	[109]
	5 µM	IMR-32 human neuroblastoma cell line treated with MeHg	Upregulation of Nrf2 and metallothionein; Downregulation of p53, bax, cytochrome C, and caspase 3; Reduction of cellular oxidative stress; Maintenance of glutathione, superoxide dismutase, glutathione-S-transferase, and catalase.	[110]
	10–50 mg/kg	Rats exposed to stress during days 14–20 of pregnancy	Activation of Nrf2; Increased levels of HO-1 and mitochondrial function; Reduction of oxidative stress	[112]
Tyrosol	10–30 mg/kg	Transient, middle cerebral artery occlusion rat model	Reduced infarct volume; Improved neurological outcome	[114]
	0.1–50 µM	Mouse cortical neurons treated with CysDA	Protective effect against CysDA-induced neuronal injury	[115]
	50–200 µM	Catecholaminergic neuron cells treated with MPP+	Attenuation of mitochondrial dysfunction and intracellular ATP depletion; Upregulation of SOD-1, SOD-2, and DJ-1	[116]
	70–280 mg/kg	C57BL/6 male mice treated with LPS	Nrf2 activation; Improvement of HO-1	[117]
Oleuropein	50 mg/kg	TgCRND8 mice (model of amyloid-β deposition)	Improvement of cognitive performance; Reduction of β-amyloid levels and plaque deposits	[120]
	50 mg/kg	Aged TgCRND8 mice	Interference with Aβ42 and pE3-Aβ aggregation; Counteraction of glutaminylcyclase-catalyzed pE3-Aβ generation	[121]
	12.5/0.5 mg/kg	TgCRND8 mice	Improvement of cognitive functions; Aβ42 and pE-3Aβ plaque number and area were reduced in the cortex	[122]
	50–100 µM	*C. elegans* (model of AD)	Reduction of Aβ plaque deposition, toxic Aβ oligomers, paralysis; Increased lifespan	[123]
	20–25 µg/mL	PC12 cell treated with 6-OHDA	Reduction of cell damage, biochemical markers of cell death	[124]
	1 pM	PC12 cell treated with 6-OHDA	Reduction of mitochondrial ROS production; Blockage of SOD activity	[125]
	1–10 µM	HT-22 hippocampal neuronal cells	Inhibition of the translocation of mitochondrial apoptosis-inducing factor to the cytoplasm; Modification in the glutamate-induced mitochondrial dynamic imbalance; Reduction of the number of cells with fragmented mitochondria	[126]
	1–10 µM	Endothelial progenitor cells (CD31^+^/VEGFR-2^+^)	Increment in proliferation and telomerase activity; Reduction in senescent cells and ROS levels; Restoration of migration, adhesion, and tube formation of endothelial progenitor cells; Activation of Nrf2; Increment in HO-1 levels	[127]
	60 mg/kg	Hypertensive rats	Reduction of blood pressure, pro-inflammatory cytokines, and the expression of components of the renin-angiotensin system; Increase in the antioxidant defense system; Activation of Nrf2; Increment of NQO-1 and HO-1	[128]

Aβ: β-amyloid; DOPAL: 3,4-dihydroxyphenylacetaldehyde; GSH: glutathione; HO-1: heme oxygenase-1; 6-OHDA: 6-hydroxydopamine; LPS: lipopolysaccharide; MAO: monoamine oxidase inhibitors; NQO1: NAD(P)H quinone oxidoreductase-1; 3NP: 3-nitropropionic acid; Nrf2: nuclear transcription factor (erythroid-derived 2)-like 2; ROS: reactive oxygen species; SOD: superoxide dismutase.

**Table 2:** Wine polyphenols and their potential involvement in neurodegenerative diseases.

Compound	Dose	Model/Treatment	Results	Reference
Resveratrol	1–20 µM	Neuronal stem cells treated with oxygen-glucose deprivation/reoxygenatation	Protection against oxygen-glucose deprivation/reoxygenation; Activation of Nrf2; Upregulation of NQO-1 and HO-1; Increase in SOD and GSH; Reduction of MDA	[129]
	10 µM	HT22 neuronal cell treated with glutamate	Activation of Nrf2; Upregulation of HO-1	[130]
	10 mg/kg	Rodent astrocyte culture	Activation of Nrf2; Increase in NQO-1 levels	[131]
	15–30 mg/kg	Adult male Sprague–Dawley rats with cerebral ischemic injury	Activation of Nrf2; Upregulation of HO-1; Reduction of oxidation biomarkers; Reestablished SOD activity	[132]
	20 mg/kg	Rat model of rotenone-induced PD	Amelioration of rotenone-induced endoplasmic reticulum stress; Restored redox balance; Activation of Nrf2; Activation of glutathione peroxidase	[133]
	100 mg/kg	Postnatal day 7 rat pups with fetal alcohol spectrum disorders	Prevention of ethanol-induced apoptosis; Scavenging ROS; Increment of survival cerebellar granule cells; Restored ethanol-induced changes of Nrf2; Retained the expression and activity of NQO1 and SOD in cerebellum	[134]
	2.4 g/kg	Coronary arterial endothelial cells/ mice fed a high-fat diet	Activation of Nrf2; Upregulation of its driven antioxidant enzymes; Restoration of normal endothelial function; Reduction of ROS production	[137]
	100 mg	Healthy volunteers	Activation of Nrf2; Expression of NQO-1 and GST-P1; Reduction of pro-inflammatory biomarkers	[139]
	500 mg/day	Non-dialyzed CKD patients	No difference in antioxidant or anti-inflammatory response	[140]
Epicatechin/Catechin	100 nM	Cortical astrocytes	Activation of Nrf2; Upregulation of GSH	[141]
	100 µM	Embryonic cortical neuronal cells	Reduction of oxidative stress; Promotion of cell viability	[142]
	30 mg/kg	Brain ischemic injury mouse models	Reduction size of damage and neurological deficits; Improvement of consequences of the brain infarct after injury; Activation of Nrf2; Increment of HO-1	[142]
	10–40 mg/kg	Transient middle cerebral artery occlusion rats	Restoration of Nrf2; Increased expression of HO-1, GSTα, and NQO1	[143]
	60 mg/mL	Zebrafish model prone to developing tau aggregation	Reduction in the formation of tau aggregates; Activation of Nrf2; Suppression of neurotoxicity	[144]
Quercetin	25 µM	Primary cerebellar granule neurons treated with H_2_O_2_ provoking oxidative insult	Activation of Nrf2; Prevention of neuronal death; Increase of GSH and GCLC levels	[146]
	50 mg/kg	Mouse model for traumatic brain injury	Translocation of Nrf2 to the nucleus	[147]
	20–50 mg/kg	Mice treated with D-galactose to induce neurotoxicity	Improvement of learning and memory; Prevention of changes in neuronal cell morphology and apoptosis in the hippocampus; Activation of Nrf2; Increased HO-1 and SOD levels	[148]

CKD: chronic kidney disease; GCLC: glutamate-cysteine ligase catalytic; GSH: glutathione; GST: glutathione S-transferase; HO-1: heme oxygenase-1; MDA: malondiadehyde; NQO1: NAD(P)H quinone oxidoreductase-1; Nrf2: nuclear transcription factor (erythroid-derived 2)-like 2; PD: Parkinson disease; ROS: reactive oxygen species; SOD: superoxide dismutase.
